# Complete Genome Sequence of *Martelella endophytica* YC6887, Which Has Antifungal Activity Associated with a Halophyte

**DOI:** 10.1128/genomeA.00366-15

**Published:** 2015-05-07

**Authors:** Ajmal Khan, Haji Khan, Eu Jin Chung, Mohammad T. Hossain, Young Ryun Chung

**Affiliations:** Division of Applied Life Science (BK21 Plus), Plant Molecular Biology and Biotechnology Research Center, Gyeongsang National University, Jinju, South Korea

## Abstract

*Martelella endophytica* YC6887, which produces antifungal compounds against fungal and oomycete pathogens, was isolated from the root of a halophyte, *Rosa rugosa*, collected at a tidal flat in South Korea. Its full-genome sequence shows that it is a circular DNA, without a plasmid, of about 4.8 Mb in size.

## GENOME ANNOUNCEMENT

The genus *Martelella* was first described as a novel genus in 2005, with only one type species, *Martelella mediterranea*, isolated from a lake (Martel) in Spain (1). *Martelella endophytica* strain YC6887 was isolated from a halophyte, *Rosa rugosa*, collected at a tidal flat in South Korea and identified as a novel species in the genus *Martelella* on the basis of 16S rRNA gene sequence analysis and biochemical and physiological characteristics (2).

The strain *M. endophytica* YC6887 was found to have antimicrobial activity (3). For the complete genome sequencing of *M. endophytica* YC6887, high-quality genomic DNA was extracted with the blood and cell culture DNA minikit (Qiagen, MD), and its complete genome was sequenced and characterized by ChunLab, Inc. (Seoul, South Korea) using an Illumina GA IIx sequencer (San Diego, CA) with 100-bp paired-end libraries. For the assembly of all sequence data, GS Assembler 2.3 (Roche Diagnostics, Branford, CT) and CodonCode Aligner 3.0 were used, and the gaps between all the contigs were filled by PCR amplification. Glimmer 3.02 was used to predict the coding sequences (CDSs). tRNAs were determined by tRNAscan-SE (4), while the rRNA was located by using HMMER with EzTaxon-e rRNA profiles (5, 6). The indicated CDSs were compared by rpsBLAST and NCBI reference sequences (Ref-Seq) to catalytic families (Catfam) and the NCBI COG. The SEED database was employed to determine the functional annotation of the CDSs (7, 8).

The full-genome sequence analysis of strain *M. endophytica* YC6887 brought out that its genome size is 4.8 Mb, with a single circular DNA. Its genome has no plasmid. The *M. endophytica* genome consists of a total 4,663 open reading frames (ORFs). It consists of a total of 9 rRNAs and 51 tRNAs. The G+C content is 62.14%.

### Nucleotide sequence accession number.

The complete genome sequence of *M. endophytica* YC6887 is deposited at GenBank under the accession no. CP010803.

## References

[1] Rivas R, Sánchez-Márquez S, Mateos PF, Martínez-Molina E, Velázquez E 2005 *Martelella mediterranea* gen. nov., sp. nov., a novel α-proteobacterium isolated from a subterranean saline lake. Int J Syst Evol Microbiol 55:955–959. doi:10.1099/ijs.0.63438-0. 15774691

[2] Bibi F, Chung EJ, Khan A, Jeon CO, Chung YR 2013 *Martelella endophytica* sp. nov., an antifungal bacterium associated with a halophyte. Int J Syst Evol Microbiol 63:2914–2919. doi:10.1099/ijs.0.048785-0. 23355694

[3] Bibi F, Yasir M, Song G, Lee S, Chung YR 2012 Diversity and characterization of endophytic bacteria associated with tidal flat plants and their antagonistic effects on oomycetous plant pathogens. Plant Pathol J 28:20–31. doi:10.5423/PPJ.OA.06.2011.0123.

[4] Lowe TM, Eddy SR 1997 tRNA scan-SE: a program for improved detection of transfer RNA gene in genomic sequence. Nucleic Acids Res 25:955–964. doi:10.1093/nar/25.5.0955. 9023104 PMC146525

[5] Kim OS, Cho YJ, Lee K, Yoon SH, Kim M, Na H, Park SC, Jeon YS, Lee JH, Yi H, Won S, Chun J 2012 Introducing Ez-Taxon-e: a prokaryotic 16S rRNA gene sequence database with phylotypes that represent uncultured species. Int J Sys Evol Microbiol 62:716–721. doi:10.1099/ijs.0.038075-0. 22140171

[6] Eddy SR 1998 Profile hidden Markov models. Bioinformatics 14:755–763. doi:10.1093/bioinformatics/14.9.755. 9918945

[7] Overbeek R, Begley T, Butler RM, Choudhuri JV, Chuang HY, Cohoon M, Crecy-Lagard V, Diaz N, Disz T, Edwards R, Fonstein M, Frank ED, Gerdes S, Glass EM, Goesmann A, Hanson A, Iwata-Reuyl D, Jensen R, Jamshidi N, Krause L, Kubal M, Larsen N, Linke B, McHardy AC, Meyer F, Neuweger H, Olsen G, Olson R, Osterman A, Portnoy V, Pusch GD, Rodionov DA, Ruckert C, Steiner J, Stevens R, Thiele I, Vassieva O, Ye Y, Zagnitko O, Vonstein V 2005 The subsystems approach to genome annotation and its use in the project to annotate 1000 genomes. Nucleic Acids Res 33:5691–5702. doi:10.1093/nar/gki866. 16214803 PMC1251668

[8] Yu C, Zavaljevski N, Desai V, Reifman J 2009 Genome-wide enzyme annotation with precision control: catalytic families (CatFam) databases. Proteins 74:449–460. doi:10.1002/prot.22167. 18636476

